# How Often Do Comparative Randomised Controlled Trials in the Field of Eczema Fail to Directly Compare the Treatments Being Tested?

**DOI:** 10.3390/jcm4061312

**Published:** 2015-06-17

**Authors:** Sonia Ratib, Sally R. Wilkes, Helen Nankervis, Kim S. Thomas, Hywel C. Williams

**Affiliations:** Centre of Evidence Based Dermatology, King’s Meadow Campus, University of Nottingham, Nottingham NG7 2RN, UK; E-Mails: sally.wilkes@nottingham.ac.uk (S.R.W.); helen.nankervis@nottingham.ac.uk (H.N.); kim.thomas@nottingham.ac.uk (K.S.T.); hywel.williams@nottingham.ac.uk (H.C.W.)

**Keywords:** atopic eczema, randomised controlled trials, between-group analysis, parallel design

## Abstract

The objective of the study was to identify all parallel design randomised controlled trials (RCTs) comparing treatments for eczema in recent dermatology literature that have failed to report a between-group analysis. The GREAT database (www.greatdatabase.org.uk) was searched to identify parallel group RCTs comparing two or more interventions published in the English language in the last decade, 2004 to 2013. The primary outcome was the number of studies that had not reported a between-group analysis for any of the outcomes. Where possible we re-analysed the data to determine whether a between-group analysis would have given a different conclusion to that reported. Out of a total of 304 RCTs in the study period, 173 (56.9%) met the inclusion criteria. Of the 173 eligible studies, 12 (6.9%) had not conducted a between-group analysis for any of the reported outcomes. There was no clear improvement over time. Five of the eight studies that were re-analysed yielded non-significant between-group differences yet reported significant within-group comparisons. All but one of the 12 studies implied that the experimental intervention was successful despite not undertaking any between-group comparisons. Although the proportion of all RCTs that fail to report an appropriate between-group analysis is small, the fact that any scientist who purports to compare one treatment against another then chooses to omit the key comparison statistic is worrying.

## 1. Introduction

Well-designed randomised controlled trials (RCTs) are powerful sources of evidence and are considered the most reliable way to determine whether an intervention is safe and effective [1]. Based on a search of MEDLINE, an average of 35,500 RCTs per year have been published over the last three years. Correct reporting of results from RCTs is important to inform meta-analyses in systematic reviews, which are often regarded as the highest level of evidence for the effects of healthcare [2,3].

As with many other medical fields, the dermatology research community relies on RCTs as the key method to evaluate treatment superiority, non-inferiority or equivalence. Quality of trial reporting has previously been evaluated in specific dermatology journals [4,5]. Specifically, in the last fifteen years more than 500 RCTs were conducted worldwide to identify effective treatment for this debilitating condition [6]. Therefore the quality of reporting of RCTs in the area of eczema provides a good exemplar that is likely to be generalisable to other skin conditions.

Most RCTs are designed to compare one intervention with another. In such studies, the single most important test statistic is the difference in responses between the groups. Whilst this may seem obvious, it has been our observation whilst conducting various systematic reviews for skin treatments that some researchers only report within-group analyses, measuring the change across the study period in each individual group but not between the groups. This approach results in a failure to test the key hypothesis, and may lead to misleading conclusions, if claims are made about the efficacy of treatments in the absence of the key comparisons. 

In order to establish how common this practice is, and to try and determine whether improvements in reporting guidelines mean that this major analysis error is now a thing of the past, we searched the GREAT database (www.greatdatabase.org.uk) and identified all of the eczema RCTs in the last ten years that failed to report a between-group analysis. The main conclusion of the study is that 12 (6.9%) of 173 eligible trials had not conducted a between-group analysis for any of the reported outcomes. There was no clear improvement over time. Five of the eight studies that were re-analysed yielded non-significant between-group differences yet reported significant within-group comparisons.

## 2. Materials and Methods

### 2.1. Selection of Publications

The Global Resource for EczemA Trials (GREAT) database was searched to identify RCTs of treatments for eczema. The GREAT database (www.greatdatabase.org.uk) contains records of all RCTs of treatments for established eczema published since the inception of the MEDLINE (1966) and EMBASE (1980), the Cochrane Library and the Skin Group Specialised Register databases plus the Cumulative Index to Nursing and Allied Health Literature (CINHAL), Allied and Complementary Medicine Database (AMED) and Literatura Latino Americana em Ciências da Saúde (LILACS) databases from the year 2000 onwards. A search limited to full paper, English language, parallel group RCTs comparing two or more interventions in the last decade (2004 to 2013) was conducted. We excluded cross-over and bilateral/within-person study designs as the statistical methods required are more complex than that for conventional parallel RCTs. Trial protocols were excluded as results were unavailable, and abstracts were not included due to the very limited nature of the information contained within them.

### 2.2. Review of Papers

We defined appropriate between-group analysis methods of continuous and categorical data as summarised in [Table 1]. Two independent medical statisticians (SR and SW) reviewed all the articles and identified those that had not reported a between-group analysis for any of the outcomes. Any discrepancies between the statisticians were resolved by a third person (HN). For studies that failed to test their hypothesis correctly, we explored the following characteristics: size of trial, year of publication, funding body, impact factor of journal, number of times work was cited and inclusion in a Cochrane Systematic review. We compared trial size with that of trials that did test their hypotheses. Furthermore, where possible we analysed the data to determine whether a between-group analysis would have given a different conclusion to that reported. In order to do this reanalysis, we required either the mean and standard deviation (SD) for each group for the outcomes at the end of the study, or the mean change over time and SD for each group.

**Table 1:** Appropriate statistical analysis for between-group comparison.

Outcome	Data Distribution	No. Parallel Groups	Appropriate Analysis
Continuous	Normal	2	Student *t*-test or Multivariate linear regression
	Normal	≥2	Analysis of variance or Analysis of Covariance
	Non-normal	2	Mann-Whitney U test
	Non-normal	>2	Kruskal-Wallis test
Categorical	N/A	≥2	Pearson Chi-squared test

### 2.3. Citation per Article and Journal Impact Factor

For studies that failed to test their hypothesis, the Web of Science was accessed on 26 January 2015 to determine the number of times the article had been cited. Additionally, the 2013 Journal Citation Reports journal impact factor (JIF) for the source journal was recorded.

### 2.4. Validation of the GREAT Database

All the eligible papers were manually reviewed for this study for the main outcome (between-group analysis not reported for any of the outcomes). Secondary outcomes were extracted from the GREAT database electronically: journal name, sample size, year of publication, funding body and type of study. As the latter was crucial in identifying eligible studies we decided to ensure that this information has been entered correctly in GREAT by conducting a validation. First, we took a random sample (>10%) of the excluded studies and manually searched the papers and checked that the study design corresponded with that entered in GREAT. Secondly, we checked the sample size was correctly entered in GREAT for a 10% random sample of eligible studies.

### 2.5. Abstract Results

For all studies that conducted a between-group analysis for at least one outcome, we determined how many had reported at least one between-group comparison in the paper’s abstract.

## 3. Results

### 3.1. Selection of Studies

**Figure 1:**
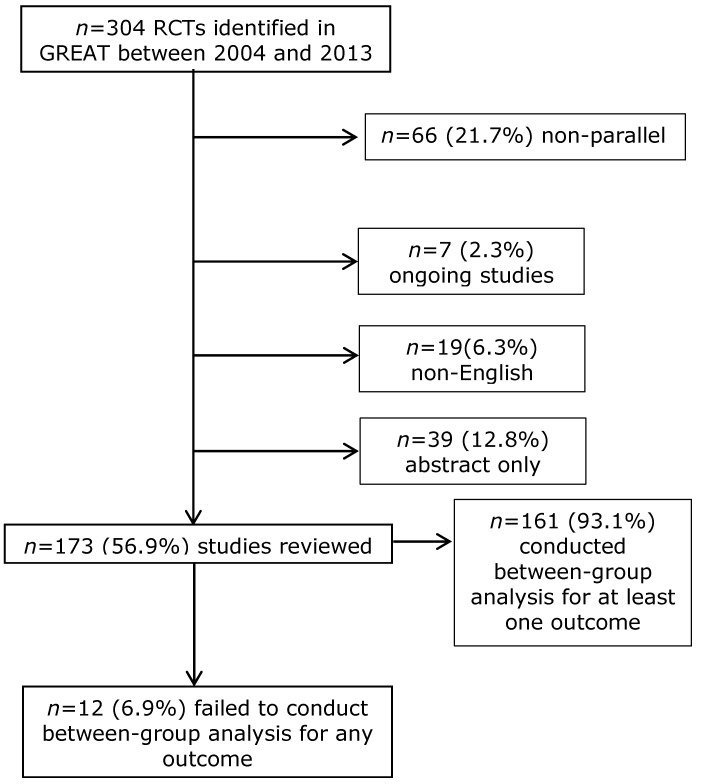
Flow chart of selected studies that failed to test hypothesis correctly.

A total of 304 RCTs conducted between 2004 and 2013 were identified in the GREAT database. Exclusions were made if the study was not of parallel design (*n* = 66), if the study was ongoing (*n* = 7), if the publication was non-English (*n* = 19) or if the publication was abstract only (*n* = 39). This left 173 (56.9%) complete studies that were eligible for review. Of the 173 studies, 12 (6.9%) failed to conduct a between-group analysis for any of the reported outcomes (Figure 1). The statisticians were unsure about the inclusion of two studies [7,8]. The third party decided that only one of these should be included [8].

**Table 2:** Studies which failed to compare the hypothesis.

Author	Title	Year	Size	Journal	Impact Factor	Citations	Funding	Conclusion
Draelos, Z [9]	Pharmacokinetics of topical calcineurin inhibitors in adult atopic dermatitis: A randomized, investigator-blind comparison	2005	37	Journal of the American Academy of Dermatology	4.91	38	Not reported	“Pimecrolimus appears to be associated with lower systemic drug exposure than tacrolimus.”
Taniuchi, S [10]	Administration of *Bifidobacterium* to infants with atopic dermatitis: Changes in fecal microflora and clinical symptoms	2005	17	Journal of Applied Research	0	10	Commercial	“Administration of bifidobacteria to infants with cow’s milk hypersensitivity with atopic dermatitis significantly increased the proportion of bifidobacteria in the fecal microflora and also might improve their allergic symptoms.”
Kimata, H [11]	Improvement of atopic dermatitis and reduction of skin allergic responses by oral intake of konjac ceramide	2006	50	Paediatric Dermatology	1.52	23	Government	“These results demonstrated that oral intake of konjac ceramide significantly improved skin symptoms in children with AD.”
Hennino, A [12]	Influence of measles vaccination on the progression of atopic dermatitis in infants	2007	12	Pediatric Allergy & Immunology	3.86	2	Commercial	“These data suggest that measles vaccination not only does not aggravate AD, but may also improve some of the immunological parameters of this allergic disease.”
Reitamo, S [13]	The pharmacokinetics of tacrolimus after first and repeated dosing with 0.03% ointment in infants with atopic dermatitis.	2009	53	International Journal of Dermatology	1.23	12	Not reported	“Treatment was well tolerated and led to considerable improvement.”
Yokoyama, Y [14]	Ethylene vinyl alcohol (EVOH) fiber compared to cotton underwear in the treatment of childhood atopic dermatitis: A Double-blind randomized study	2009	21	Indian Pediatrics	1.04	2	Not reported	“Ethylene vinyl alcohol fiber underwear might be useful for children with atopic dermatitis.”
Yoshida, Y [15]	Clinical effects of probiotic *Bifidobacterium breve* supplementation in adult patients with atopic dermatitis	2010	24	Yonago Acta medica	0.27	3	Commercial	“Our results suggest that *B. breve* may be beneficial for the treatment of atopic dermatitis.”
Byun, HJ [16]	Full-spectrum light phototherapy for atopic dermatitis	2011	38	International Journal of Dermatology	1.23	11	University	“We showed that FSL phototherapy can be an effective and safe treatment option in AD.”
Amestejani, MD [8]	Vitamin D supplementation in the treatment of atopic dermatitis: A clinical trial study	2012	60	Journal of Drugs in Dermatology	1.95	41	Not reported	“Supplementation with oral vitamin D dramatically improved disease severity in AD patients.”
Bae, B [17]	Progressive muscle relaxation therapy for atopic dermatitis: Objective assessment of efficacy	2012	25	Acta Dermato-Venereologica	4.24	19	Not reported	“Progressive muscle relaxation may be a useful adjunctive modality for the management of atopic dermatitis through the reduction of anxiety.”
Chung, BY [18]	Dose-dependent effects of evening primrose oil in children and adolescents with atopic dermatitis.	2013	40	Annals of dermatology	0.95	1	University	“The results of this study suggest that the 320 mg and 160 mg of primrose oil groups may be equally effective in treating AD patients.”
Iyengar, SR [19]	Immunologic effects of omalizumab in children with severe refractory atopic dermatitis: A randomized, placebo-controlled clinical trial	2013	8	International Archives of Allergy and Immunology	2.43	21	Non-profit organisation	“Patients on anti-IgE therapy had an improvement in clinical outcomes as measured by the SCORAD system; however, these effects were comparable to improvements in the control group.”

### 3.2. Characteristics of Studies that Failed to Compare Their Hypothesis

Table 2 displays the characteristics of the 12 studies that had not conducted a between-group analysis for any of the reported outcomes. For each year, apart from 2004 and 2008, there were either one or two studies that had failed to compare the hypothesis correctly. The interventions of three quarters of the studies were non-pharamacological [8,10,11,14,15,16,17,18].

The sample size of the trials varied from 8 to 60 participants. The median sample size was 31 and interquartile range [IQR] was 21 to 40. Only two studies had a sample size greater than 50. In contrast, the number of participants in the 161 studies that conducted a between group analysis was significantly higher (median 68 [IQR 40, 142], *p* < 0.01).

With respect to funding of the trials; three had commercial funding, four were publicly funded and for five the funding was unclear. 

A third of the studies (*n* = 4) mentioned in their statistical methods section that that they would compare groups [12,14,17,18]. One paper stated at the onset that their study had not been designed to do hypothesis testing, but rather to describe pharmacokinetic profiles and to perform secondary, non-statistical evaluations of local tolerability, safety and efficacy of the drugs. As such, the sample size for the study was not based on statistical considerations [9].

The impact scores of the 12 journals, in which the trials were published, varied from 0 to 12.25, with a third having an impact score of 2 or greater. Table 2 displays the number of citations per paper; these ranged from 2 to 41 times. The study by Amestajani* et al.* [8] had been cited the most frequently, at the time of writing. The authors investigated the effect of Vitamin D supplementation on eczema compared to placebo. Of the 41 citations, 15 (36.6%) had cited the work in such a way as to indicate that they had accepted that the study had produced positive findings. For example, one study reported “Supplementation with oral vitamin D has led to significant improvement in patients with atopic dermatitis” [20] and “Randomised control trials have evaluated the effect of supplementation with vitamin D on the severity of atopic dermatitis and all found a reduced severity of the disease in the supplemented group compared to placebo” [21]. Only one of the 41 authors had actually interpreted the results correctly and had not concluded that the intervention was effective; they write “The RCT showed a significant improvement of AD severity for subjects assigned to vitamin D, but only when compared with their own baseline severity, not the randomly assigned controls” [22]. 

Of the 12 studies that failed to conduct a between-group analysis, one had been included in a Cochrane Skin review [10]. The authors of the Review reported there was no adequate between-group comparison in this study and the data could not be used in the meta-analysis [23].

### 3.3. Misleading Conclusions 

The conclusions of the 12 potentially misleading articles are summarised in Table 2. All authors except one implied that the intervention could be an effective treatment for people with eczema in their conclusions. 

Out of the 12 articles, we were able to conduct between-group analyses using data, presented in the papers, from eight (Tanuichi *et al.* [7], Amestejani *et al.* [8], Reitamo *et al*. [13], Yokoyama *et al.* [14], Yoshida* et al.* [15], Bae* et al.* [17], Chung* et al.* [19] and Iyengar* et al.* [21]) and six yielded non-significant results for the appropriate analysis[7, 14, 15, 17, 19, 21]. All, apart from the study by Iyengar* et al.*, reported significant within-group differences. For example, Yokoyama* et al.* concluded that “objective SCORAD and urinary cortisol levels (*p *< 0.05) were decreased only in the EVOH group”. This is a potentially misleading statement which implies that EVOH is effective, yet there was a non-significant difference between the two groups. Yoshida* et al. *stated in the abstract that “in the quality of life assessment, the total score showed significant improvement in the probiotic group”. The authors did not mention the placebo group or any comparison of the two groups. However, the authors did acknowledge that the two groups were significantly different in eczema severity levels at baseline. Bae* et al.* did acknowledge in the abstract that whilst there was a significant effect in the intervention group for EASI, this effect was also seen in the control group. Chung* et al.* stated in the abstract that “the improvement in EASI scores was greater in the 320 mg group than in the 160 mg group” without comparing the two groups directly. Tanuichi* et al.* stated in the abstract that the proportion of Bifidobacterium in the bifidobacteria-administered group increased after 3 months whilst there were no significant changes in the control group. This is potentially misleading as there was no statistically significant difference between the two groups. Iyengar* et al.* reported raw data for each of the eight patients in the study and did not make claims on the significance of the findings. Analysing the differences in SCORAD for this trial produced a borderline significant result (0.0557) in favour of the placebo group. 

There were a number of reasons why the remaining four papers could not be re-analysed such as failure to report measures of variance and missing raw data values. Draelos* et al.* [9] did go some way to justifying the lack of a between-group comparison by explicitly stating that the study was under powered to carry out hypothesis testing but rather to describe pharmacokinetic profiles and to perform secondary, non-statistical evaluations of local tolerability, safety and efficacy of pimecrolimus and tacrolimus.

### 3.4. Validation of the GREAT Database

Of the 66 non-parallel studies that were excluded from the analysis, we randomly selected 10 and checked that the study design entered in the GREAT database matched what was reported in the paper. Similarly of the 161 that had conducted a between-group analysis correctly, 10% (*n* = 17) were randomly checked to validate sample size. In all cases, what was entered in GREAT database corresponded with what was written in the papers.

### 3.5. Exploration of Abstracts for Studies that Had Conducted a Between-Group Analysis

We read the abstracts of the 161 papers that had conducted a between-group analysis for at least one outcome. However, it was not possible to quantify the number of studies that had reported the results of an adequate comparison in the abstract due to poor reporting and possibly word count limits. Some abstracts did not provide any quantitative information but were narrative in form, so it was unclear whether a between-group analysis had been conducted. For example, it was often reported “treatment *x* was shown to be as effective as treatment *y*”. This statement in itself is of concern, as most trials are powered as superiority trials.

## 4. Discussion

### 4.1. Main Findings

Our study has shown that the proportion of eczema parallel RCTs published between 2004 and 2013 that did not compare the groups appropriately was 7%, and was similar over the entire study period demonstrating that the error is an ongoing issue. There was no strong association with type of funding body or journal. The sample size of each of the studies was relatively small (median size 31). One paper was clearly described as a pilot study, and only one group of authors explicitly stated that they never had any intention of examining between-group differences. All but one of the studies implied that the experimental treatment/intervention was successful despite failing to make any between-group comparisons.

The phenomenon of avoiding between-group analysis that we have identified here, could be described as “selective analysis reporting bias”. Possible explanations for this bias include researchers conducting a between-group analysis which reveals no statistically significant findings but then deliberately concealing such a finding to “save face” and to enhance the within-group changes in the hope that journals are more likely to accept their paper for publication. Another reason may be that they are pilot studies with no intention of testing the between-group differences as was the case in 1of the 12 studies we found. We would argue that between-group analyses should always be presented in a study that compares one treatment against another as even small studies can contribute to a meta-analysis and to sample size calculations of future studies. Other reasons which we speculate may lead to “selective analysis reporting bias” (but are hard to quantify) are an innocent lack of understanding by the researchers of the basic principles of comparing two treatments, or not having the submitted workpeer-reviewed by a statistician or a dermatologist with clinical trial skills. Although the proportion of all RCTs that fail to report appropriate between-group analysis for *any *outcome is small, the fact that any scientist planning to compare two groups omits the key comparison statistic is worrying.

Taking into account that our definition of “failed hypothesis” was a generous one, that is, only those with none of the outcomes compared correctly, we are aware that the degree of “selective analysis reporting bias” is probably greater than reported here. A total of 161 eligible studies carried out between-group analyses for at least one outcome. They may not have analysed all reported outcomes due to “selective analysis reporting bias”. The phenomenon of “selective reporting outcome bias”, which is where one or more outcomes have either been omitted, changed or introduced compared to what was originally planned in the trial protocol [24,25], is also likely to be a contributory factor to even more papers failing to present the results as per the original outcome. Further research is therefore warranted to explore the extent of “selective analysis reporting bias” and its association with “selective reporting outcome bias”.

### 4.2. Strengths and Limitations

The study used the GREAT database to identify all eczema RCTs published over the last decade. Using this global resource allowed for the time-efficient and cost-effective completion of this review. Including all trials on a particular topic, rather than just those reported in specific journals as others have done [4,5], or as a random sample of published trials, reduces the risk of selection bias and makes the study comprehensive and representative of worldwide eczema trials, thus increasing the external validity of this study. It is possible that for other skin conditions, the proportion of studies that have failed to test their hypothesis correctly may be different, which renders the results of this study difficult to generalise to the whole field of dermatology, or indeed medicine.

Our validation of the GREAT database demonstrated that the data extracted were reliable and consistent with the original papers. Given that two statisticians reviewed the eligible papers independently, and a third party resolved any discrepancy, we made every effort to ensure that these findings are accurate. The analysis required for cross-over studies and bilateral ones is more complex than that of a parallel design. Although not as common as parallel group studies, we did exclude 22% (*n* = 66) of trials as they were of different design. Given that more sophisticated analysis is required for such studies it is possible that the proportion of all trials that failed to test their hypothesis correctly is higher than 7%. 

One could argue that the exclusion of 19 non-English trials may have affected the estimate we report. The direction of this bias is hard to speculate as there is limited literature assessing the association between language of publication and trial quality. However one study, conducted in 1996, compared the completeness of reporting of trials published in English and other-language trials. Those in other languages were less likely to report a clearly pre-specified primary outcome or any rational for sample size classification [26]. The 19 papers we excluded had abstracts in English. From the information reported in the abstracts, we were able to determine that 11 of the 19 (57.9%) had conducted a between-group comparison on at least one outcome, three had not, and it was unclear what the remaining ones had done.

Finally, our re-analysis of the raw data was limited to follow-up mean differences. It was not possible to look at improvements over time or to account for baseline differences where they existed. The total number studies with incorrect conclusions could therefore not be determined.

### 4.3. Comparison with Other Studies

A study by Alvarez* et al.* [5] compared the quality of reporting pre- and post-CONSORT adoption in the Journal of American Academy of Dermatology and the British Journal of Dermatology (BJD). One of the quality criteria included adequacy of group comparison. The authors calculated the proportion of the 98 studies that had failed to conduct a between-group analysis of the primary endpoint at a single predefined time point. The proportions were 13% and 11% in 1997 and 2006 respectively; there was no significant change over time. These proportions are slightly higher than those we report here as our definition was stricter and we only included parallel design studies. Similar to our findings, there was no change over time. 

### 4.4. Implications of Our Findings

The main message of this study is that all studies that set out to compare treatments should present data that follows through in comparing those treatments with appropriate measures and confidence intervals, regardless of whether the differences are statistically significant or not. The introduction of mandatory trial registration with some journals such as the Journal of Investigative Dermatology (JID) and British Journal of Dermatology is an opportunity to improve the quality and truthfulness of statistical analyses [27,28]. A prospectively defined protocol and a statistical analysis plan made available to reviewers may reduce the prevalence of failed hypothesis testing and research wastage. Unfortunately, adequate trial registration for eczema RCTs is poor. Our study adds to the current literature that recommends the registration of trials to ensure the transparent reporting of clinical trial results [24,27]. Our report also highlights that abstracts of parallel group RCTs in eczema can be misleading in the way they fail to mention the key comparisons and how they fail to analyse them in an appropriate way. There are ethical implications of recruiting patients into trials in good faith, only to essentially “throw their data away”. Further, the costs of such failure are wasted research effort and potentially misleading results that could harm patients.

On submission of a clinical trials report to a journal, peer review should include input from a trained statistician or reviewer with good methodological expertise in clinical trials. The recent appointment of a Statistical Editor by the BJD for example demonstrates the importance journal editors are starting to place on assessing statistical analyses. Journal referees should scrutinize results rigorously to determine whether a trial has failed to answer the main hypothesis or not. The fact that the one study included in a systematic review was recognised by reviewers to have not reported between-group estimates and not included in the meta-analysis is reassuring.

Of the 12 studies that failed to test their hypothesis correctly, eight (75%) of these used non-pharmacological products like evening primrose oil and vitamin D. This probably reflects the different regulatory framework for pharmaceutical and non-pharmaceutical studies. Future work may be necessary here, to explore the association between quality of trials and the type of intervention.

Finally, awareness of the phenomenon of “selective analysis reporting outcome bias” needs to be broadcasted widely so that researchers, funders, ethics committees, editors, peer-reviewers and readers are aware of the problem.

## Conclusions

Although the proportion of all eczema RCTs that fail to report an appropriate between-group analysis is small, the fact that any study that sets out to compare two or more treatments fails to present any data on such a key comparison is worrying. This phenomenon of only presenting within-group treatment responses rather than between-group responses, represents a form of reporting bias and potential research wastage. 
